# Macroelements and heavy metals content in energy crops cultivated on contaminated soil under different fertilization—case studies on autumn harvest

**DOI:** 10.1007/s11356-018-1490-8

**Published:** 2018-02-16

**Authors:** Marta Pogrzeba, Szymon Rusinowski, Jacek Krzyżak

**Affiliations:** Institute for Ecology of Industrial Areas, 6 Kossutha Street, 40-844 Katowice, Poland

**Keywords:** *Miscanthus* x *giganteus*, *Spartina pectinata*, Heavy metals, Macronutrients, Senescence, Drought

## Abstract

Heavy metals (HMs) contamination of soils is a major problem occurring worldwide. Utility of energy crops for biofuel feedstock production systems offers a feasible solution for a commercial exploitation of an arable land contaminated with HMs. Experiments involved field testing of *Miscanthus* x *giganteus* and *Spartina pectinata* cultivated on HMs-contaminated soil with standard NPK fertilizers and commercially available microbial inoculum. Biomass yield, water content, macronutrients (N, P, K, Mg, Ca), and heavy metal (Cd, Pb, Zn) concentrations in plant shoots were assessed at the end of the first and the second growing season. Independently of the applied fertilizers, *Miscanthus* x *giganteus* produced higher biomass yield while contrary results were obtained for *S. pectinata.* Higher HMs content in plants influenced the status of the mineral macronutrients in particular N and K. Occurrence of hasted senescence induced by drought in the second growing season caused reduction in the concentrations of all elements (except Pb), due to earlier rhizomes relocation.

**Electronic supplementary material:**

The online version of this article (10.1007/s11356-018-1490-8) contains supplementary material, which is available to authorized users.

## Introduction

There are approximately 20 elements essential for plant growth and development. They can be divided into two groups: mineral macro- and micronutrients. Mineral macronutrients are required for plants in relatively high amounts in comparison with other elements. Mineral macronutrients (N, P, K, Ca, Mg, Fe, S) are divided into primary and secondary categories (Tripathi et al. 2014). Primary macronutrients represented by N, P, and K are often main components of fertilizers which are introduced to soil in different chemical forms. Both groups of macronutrients play a significant role in the metabolism of plants (protein, DNA, RNA, plant photosynthetic pigment components, enzyme cofactors associated with metabolites transport) as well as in protecting them against different abiotic stressors, including the presence of heavy metals (HMs) in the environment (Tripathi et al. 2014; Waraich et al. 2011; Nazar et al. 2012).

Heavy metals in the environment are a common plant stress factor, which can be harmful to plants as well as indirectly to humans due to their placement in the trophic chain, since plants accumulate them (Alloway 2013). Some HMs are however necessary for the development of plants, e.g., Fe, Cu, Mn, Mo, and Zn, although their presence in excess amounts can be toxic for plants. Beyond that, there are also other HMs (Cd, Hg, Pb) which are not associated with plant development and can cause damage to them (Siedlecka 2014).

The presence of HMs in the environment can have a natural (e.g., volcanic emission) and/or industrial (e.g., smelters, coal mines) origin. Industry is the main emitter of HMs to the environment, especially in regions associated with the processing of these metals. Such areas are usually highly contaminated, where the pollutants can also spread throughout the vicinity (e.g., due to dust emission) consequently influencing cultivated crops. Therefore, such arable lands should be excluded from agricultural production (Van Slycken et al. 2013). Biomass production using perennial plants with low cultivation requirements could be an alternative here. Application of such approach provides a double benefit, both in degraded land management as well as phytoremediation, due to the stabilization or extraction of toxic elements by plants (Meers et al. 2010; Balsamo et al. 2015).

*Miscanthus* x *giganteus* and *Spartina pectinata* are second-generation energy crops which are perennial grasses capable of performing C_4_ photosynthesis. It makes them much more effective in biomass production than C_3_ plants. Due to the specific development of their underground organs, both grasses are able to allocate nutrients to rhizomes during senescence (Sarath et al. 2014). Mineral composition of these plant species is very important when considering the biomass feedstock destination. Additionally, it has also been found, that *M.* x *giganteus* (Pogrzeba et al. 2010; Korzeniowska and Stanislawska-Glubiak 2015; Pogrzeba et al. 2017) and *S. pectinata* (Zhang et al. 2015; Korzeniowska and Stanislawska-Glubiak 2015) demonstrate the ability to accumulate HMs in the aboveground organs. This capability combined with a high biomass yield make these plants particularly useful for cultivation on HMs contaminated lands (Van Ginneken et al. 2007).

There are numerous literature sources addressing the relationship between HMs and mineral macro- and micronutrients. They however usually correspond to annual plants cultivated for food and medical purposes (Singh et al. 1990; Zhang et al. 2002, Bello et al. 2004; Khan and Bano 2016). Additionally, there is a lack of papers considering the effect of heavy metals on mineral nutrient status, that would refer to field tested perennial grasses cultivated for biomass feedstock on contaminated arable land (Nsanganwimana et al. 2016).

The aim of the presented study was to describe the relationship between HMs and mineral macronutrients uptake to the aboveground plant organs of *M.* x *giganteus* and *S. pectinata* cultivated in field conditions on a contaminated arable land, the changes in mineral composition of plants during their acclimatization (first two growing seasons from trial establishment), and the influence of NPK fertilizer and commercially available microbial inoculum on the plant mineral composition. Thus, we hypothesize that changing in plant mineral composition depends on the differences in HMs concentration in plant shoots caused by fertilization rather than on applied fertilizers itself.

## Materials and methods

### Site description

**Fig. 1:**
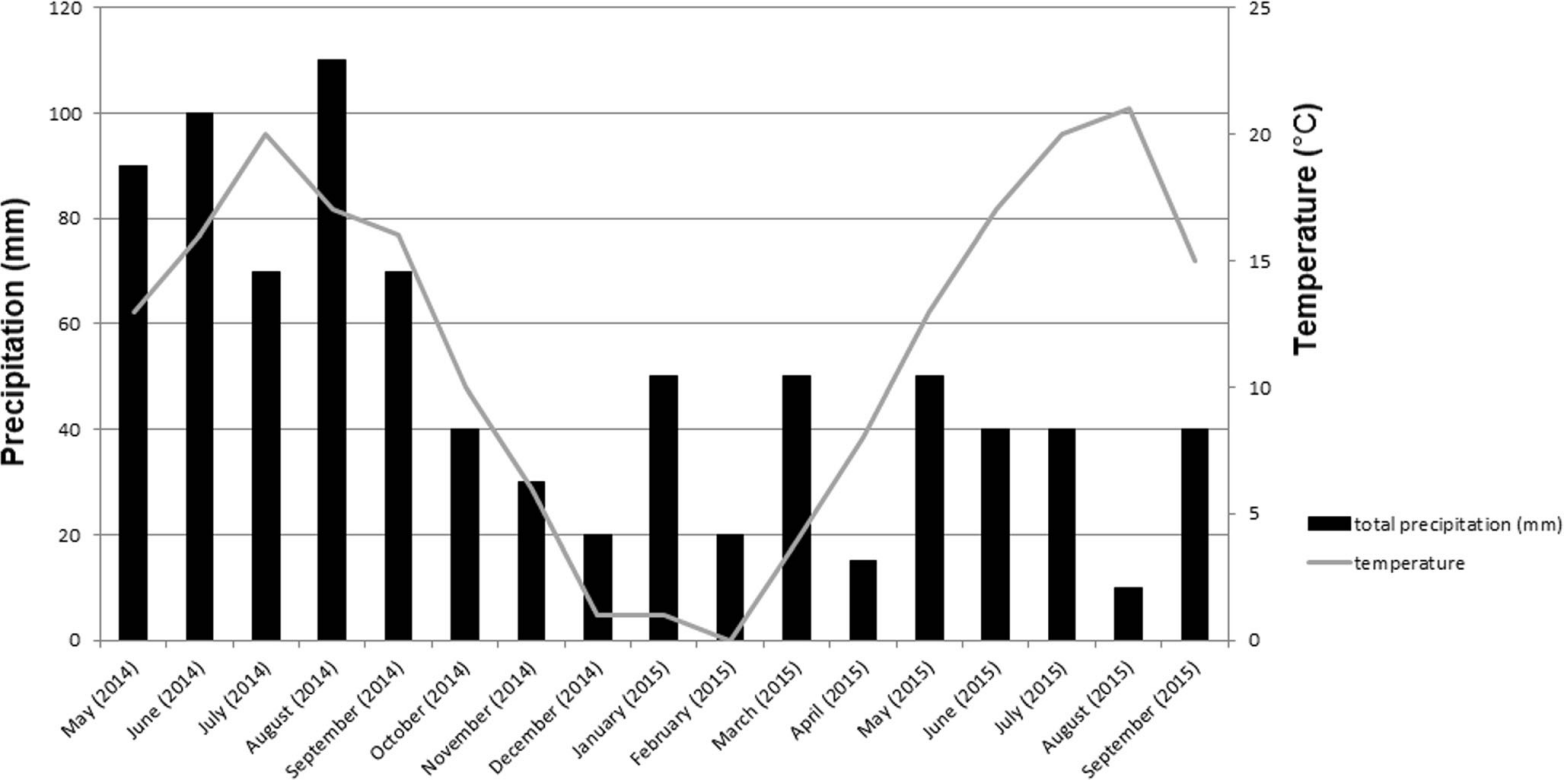
Meteorological data presented monthly total precipitation (columns) and average temperature (line) from May 2014 to September 2015

The presented data was collected at the end of the first and second growing season (September 2014 and 2015) from a HM-contaminated arable area in Bytom (Upper Silesia, Poland, 50° 20′ 43.0″ N 18° 57′ 19.6″ E) on an experimental site of the Institute for Ecology of Industrial Areas. This agricultural land was affected in the last century by dust falling from lead and zinc smelter, causing HMs soil contamination, especially zinc, cadmium, and lead. The soil concentrations of Pb, Cd, and Zn at the site exceed the limits set by Polish law (Dz.U. 2016 poz. 1395), which refers to the exclusion of such areas from food production. The climate in Poland is moderate. Average values of temperature and total precipitation measured during 2014 and 2015 growing season were 17/17 °C and 455/300 mm, respectively (Institute of Meteorology and Water Management, Fig. 1).

### Experimental design

The trial of *M.* x *giganteus* (M) and *S. pectinata* (S) was established in May 2014. For *M*. x *giganteus*, 45-g rhizomes were planted at 10-cm depths, obtained from direct cutting of rhizomes (7–10-cm length) at the beginning of the May 2014. For *S. pectinata*, root seedlings were pre-cultivated in greenhouse conditions. All planting materials were obtained from uncontaminated sites. On each plot, 49 plants were planted over an area of 16 m^2^ (3 plants per 1 m^2^) with a buffer zone of 4 m between each plot, which protected plants against uncontrolled fertilization. Single plot trials with pseudo-replication were used due to apprehension of uncontrolled widespread of microbial inoculum during foliar application.

Each plot was treated in a different way:

- Control (without treatment);
- NPK standard fertilization—applied directly to the soil before planting (*M*. x *giganteus*—nitrogen 70 kg ha^−1^, phosphorus 30 kg ha^−1^ as P_2_O_5_, and potassium 45 kg ha^−1^ as K_2_O; *S. pectinata*—nitrogen 80 kg ha^−1^, phosphorus 50 kg ha^−1^ as P_2_O_5_, and potassium 75 kg ha^−1^ as K_2_O), using commercially available fertilizers; Polifoska (Grupa Azoty, Zakłady Chemiczne “Police” S.A., Poland: N—4% as NH_4_; P_2_O_5_—22%; K_2_O—32%; MgO—2%; SO_3_—9%) and ammonium nitrate (PULAN® 34 N, Grupa Azoty Zakłady Azotowe “Puławy” S.A., Poland: NH_4_—17%; NO_3_—17%)
- Commercial microbial inoculum—Emfarma Plus® ProBiotics Poland (lactic acid bacteria > 3.0 × 10^5^ cfu ml^−1^, yeast < 1.0 × 10^6^ cfu ml^−1^, and purple non-sulfur bacteria > 1.0 × 10^4^ cfu ml^−1^ in molasses suspension). Eight liters of 10% water solution of Emfarma Plus® was sprayed on the soil surface; additionally, the roots of the seedlings were soaked in this solution at the beginning of the experiment. Plant leaves were treated monthly during the growing season with 10% water solution of Emfarma Plus® as aerosol treatment (8 l per plot)

### Plant material sampling

**Fig. 2:**
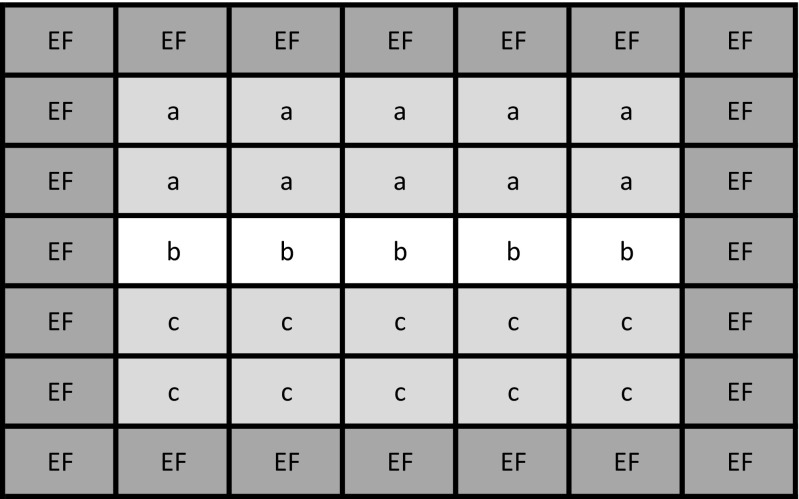
Scheme of plots division in the experiment. EF—plants exposed to edge effect. a, b, c—section where samples were collected

The data for further analysis was collected from plots divided into three sections. Within sections (a) and (c), two plants were selected, while within section (b), one plant was selected at random. Plots were divided into sections to obtain adequate sample randomization. For each of the randomly selected plants, stems were harvested for further analysis (Fig. 2).

### Soil characteristic and biomass elemental analysis

Soil pH was measured in H_2_O (ratio 1:2.5 *m*/*v*) with a combination of glass/calomel electrode (OSH 10-10, METRON, Poland) and a pH-meter (CPC-551, Elmetron, Poland) at 20 °C. The electrical conductivity was determined by an ESP 2ZM electrode (EUROSENSOR, Poland) according to the Polish norm PN-ISO 11265:^1997^.

Soil texture was evaluated by the hydrometric method according to the Polish norm PNR-04032:^1998^.

Soil organic matter content (OM) was measured by loss on ignition as follows: air dry soil was dried at 105 °C for 24 h and then (5 g) treated with 550 °C for 4 h (Pogrzeba et al. 2017). Soil organic carbon (C_org_) was assessed according to PN-ISO 14235:^2003^, sulfur content was assessed according to PN-EN 14582:^2017^.

The total content of metals in the soil and plant tissues was obtained using hot plate digestion (HNO_3_ and HClO_3_, at ratio of 4:1) and flame atomic absorption spectrometry (SpektrAA 300, Varian INC., USA). Soil samples were digested in aqua regia according to the ISO 11466:^1995^, while plant samples were digested in nitric and perchloric acid (4:1 *v*/*v*) (Schierup and Larsen 1981). Total nitrogen concentration in soil was measured using dry combustion method according to norm ISO 13878:^1998^. Available phosphorus and available potassium concentrations were assessed according to the method described by Egner et al. (1960). Total nitrogen concentration (N) in plant leaves was measured using the titration method (Bremner 1996), whereas total phosphorus (P) and potassium (K) concentration in plant leaves was assessed in the previously mineralized samples using ICP (Liberty 220, Varian, USA).

### Biomass production

Total biomass production was assessed at the end of each growing season. The collected plant material was dried at 70 °C, weighed, and the biomass yield of dry weight per square meter was calculated.

### Plant visual observation

Photographic documentation was collected once per month during each of the growing seasons. For this paper, September photos were described as most valuable for data analysis due to visible differences in plant development between the first and the second growing season.

### Statistical analyses

Data was analyzed using a three-way ANOVA, followed by a post hoc comparison using the Fisher LSD test (*P* < 0.05). Principal component analysis (PCA) was performed on a correlation matrix to detect any relationship between accumulation of analyzed elements and water content. Additionally, PCA was used to distinguish cases clusters created on two principal component (PC) axes. All data statistical analyses were performed using Statistica 10 (Statsoft, USA). Spider charts where constructed using Excel MS Office (Microsoft, USA), and standardization of data used for charts construction were performed using the Statistica 10 Software (Statsoft, USA).

## Results

### Soil characteristic

Soil physicochemical characteristics are presented in Table 1.

**Table 1:** Initial physicochemical soil parameters obtained from experimental plots before trial establishment

	Parameters														
	pH _H2O_	pH _KCl_	EC (μS/cm^3^)	OM (%)	C_ORG_ (%)	N_TOTAL_ (%)	S (mg kg^−1^)	Pb (mg kg^−1^)	Cd (mg kg^−1^)	Zn (mg kg^−1^)	Fe (mg kg^−1^)	Mg (mg kg^−1^)	Ca (mg kg^−1^)	P (mg kg^−1^)	K (mg kg^−1^)
Variants															
M-C	6.93 ± 0.03a	6.50 ± 0.03a	117.55 ± 1.52a	5.36 ± 0.06a	2.30 ± 0.05a	0.17 ± 0.00a	LOQ	535.10 ± 5.35a	20.56 ± 0.26a	1868.33 ± 51.83a	11.851.67 ± 31.93a	2713.33 ± 10.14c	6935.67 ± 67.86a	1238.43 ± 9.30a	1202.43 ± 14.77a
M-NPK	6.71 ± 0.06b	6.15 ± 0.03bc	93.23 ± 1.30b	5.45 ± 0.06a	2.17 ± 0.02ab	0.17 ± 0.02a	LOQ	531.97 ± 4.83a	20.81 ± 0.21a	1953.33 ± 58.40a	12.007.33 ± 65.49a	3129.00 ± 84.11b	6892.33 ± 5.78a	1216.33 ± 16.84a	1184.77 ± 6.02a
M-INC	6.80 ± 0.04ab	6.22 ± 0.03b	113.22 ± 8.11a	5.50 ± 0.06a	2.21 ± 0.04ab	0.18 ± 0.00a	LOQ	532.37 ± 4.03a	21.07 ± 0.75a	2041.67 ± 29.73a	12.197.67 ± 352.96a	4910.00 ± 88.93a	6964.33 ± 238.76a	1245.10 ± 50.05a	1209.97 ± 36.35a
S-C	6.58 ± 0.07b	6.12 ± 0.01c	86.16 ± 1.96bc	4.00 ± 0.01b	2.29 ± 0.16a	0.12 ± 0.00a	LOQ	372.50 ± 2.20b	13.97 ± 0.17b	1329.33 ± 23.10b	9317.67 ± 52.17b	1645.67 ± 26.36d	2778.00 ± 23.46b	660.80 ± 6.45b	986.67 ± 30.39b
S-NPK	6.61 ± 0.03b	6.12 ± 0.01c	83.52 ± 2.47bc	4.10 ± 0.06b	1.99 ± 0.12b	0.13 ± 0.00a	LOQ	362.77 ± 3.13b	13.60 ± 0.09b	1288.33 ± 9.60b	9244.67 ± 172.45b	1635.67 ± 28.20d	2826.00 ± 14.01b	721.33 ± 7.49b	951.83 ± 20.56b
S-INC	6.55 ± 0.07b	6.09 ± 0.02c	78.70 ± 1.55c	4.23 ± 0.03b	1.73 ± 0.07b	0.13 ± 0.01a	LOQ	366.97 ± 2.58b	13.64 ± 0.09b	1299.00 ± 6.66b	9585.67 ± 230.43b	1692.33 ± 11.85d	2831.67 ± 5.24b	739.37 ± 12.32b	995.27 ± 25.24b

Values are means ± SE. A lower case letter (a, b, c, d) denotes significant differences among soils samples taken from different plots at *P* ≤ 0.05 according to the Fisher LSD test. Every measurement was performed in three replicates (*n* = 3)

*M Miscanthus* x *giganteus*, *S Spartina pectinata*, *C* control, *NPK* NPK fertilizer, *INC* microbial inoculum

Each of the analyzed parameters in the initial soil samples was slightly lower for *S. pectinata* plots when compared with *M*. x *giganteus* plots. Heterogenic distribution of elements could be caused by irregular dust fall emitted by the smelter and unequal distribution of agricultural treatments in the past. No statistically significant differences were found for the analyzed soil parameters among *S. pectinata* plots, with the exception of C_ORG_ for plot S-C. Contrary to C_ORG_, Mg content was different for each of the *M.* x *giganteus* plots. It was found that Mg concentration at the M-NPK plot was higher by 15% although the highest value was measured at 81% for the M-INC plot in comparison to the control. It can be assumed that among species, soil parameters were highly homogenous.

### Heavy metals and mineral macronutrients plant concentrations (spider charts and PCA)

**Fig. 3:**
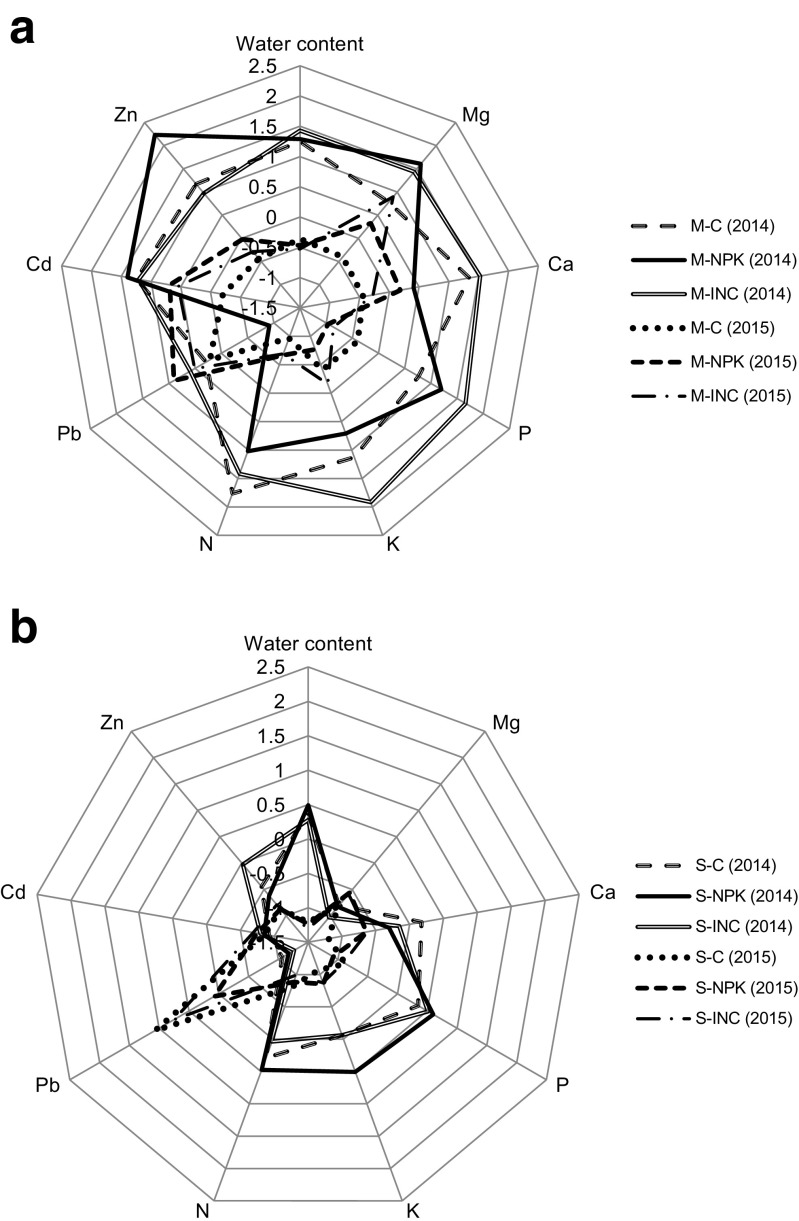
Spider charts constructed on macronutrients, heavy metals, and water content show patterns indicated changes of those parameters; among treatment (*C* control, *NPK* NPK fertilizer treated plant, *INC* microbial inoculum treated plant) and growing season (*14*—growing season 2014, *15*—growing season 2015) for: **a** *Miscanthus* x *giganteus* (M) and **b** *Spartina pectinata* (S). Mg, Ca, P, K, N, Pb, Cd, Zn—elements accumulation in aboveground plants organs. For better data visualization, all presented values were standardized. Every measurement was performed in 5 replicate (*n* = 5)

Spider charts (Fig. 3a, b) are an easy way to visualize patterns in feature characteristics of plant organisms. The presented spider charts can be divided in to three sections: heavy metal (Zn, Cd, Pb) accumulation, primary mineral macronutrients (N, K, P), and secondary mineral macronutrients (Mg, Ca) with water content. For both cultivated species, overall decrease in all parameters (except Pb) was visible after the second growing season, in comparison to the first one.

**Fig. 4:**
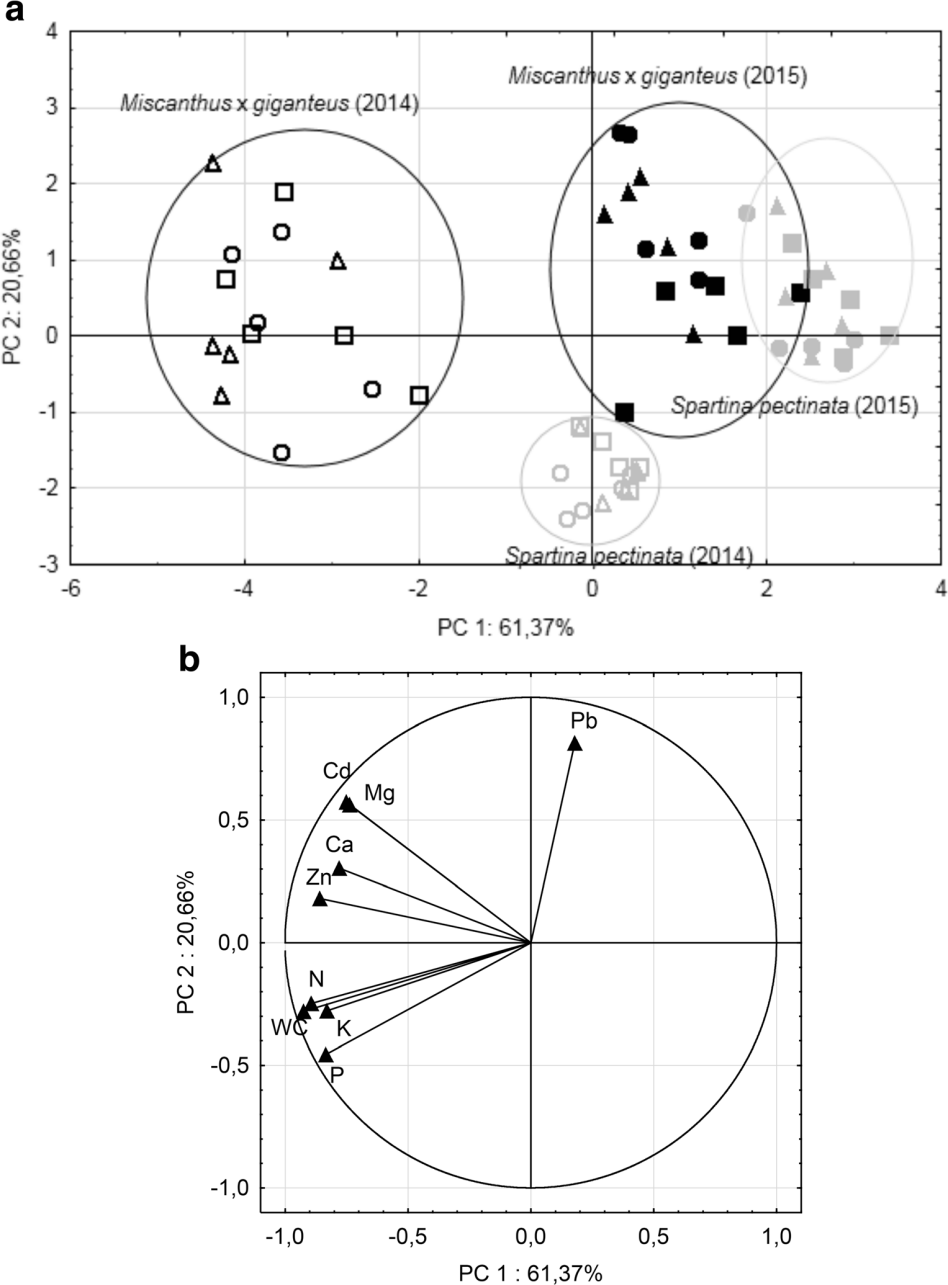
Principal component analysis distinguished into two parts: ordination of case along two PCA axis (PC1 x PC2) (**a**) and correlation between variables along two PCA axis (PC1 x PC2) (**b**) black and gray geometric figures representing *Miscanthus* x *giganteus* and *Spartina pectinata*, respectively. Open and close geometric figure representing 2014 and 2015 growing season, respectively; □—control plants; ○—NPK fertilizer-treated plants; Δ—microbial inoculum-treated plants; N, P, K, Zn, Ca, Cd, Mg, and Pb—elements concentration in aboveground plant organs; WC—water content in biomass

Principal component analysis (Fig. 4a, b) shows the multivariate relationships between the plants’ ability to accumulate HMs, primary mineral macronutrients, secondary mineral macronutrients, and, additionally, plant water content. The analyzed cases (Fig. 4a) were divided by PC1 and PC2 axis into four groups, which corresponded to the species and the growing seasons (*M.* x *giganteus* growing season 2014, *M.* x *giganteus* growing season 2015, *S. pectinata* growing season 2014, *S. pectinata* growing season 2015). The distance between the clusters indicates the differences in the analyzed parameters within the groups. The distance between *M.* x *giganteus* and *S. pectinata* after the first growing season is larger than after the second. The differences in the distances within the tested species after the first and second growing season are similar. PCA showed that the treatments are not differentiated into subgroups within the growing season and the tested plant species. Variables (Fig. 4b) can be divided into three groups of parameters: primary macronutrients with water content (1), HMs (except Pb) with secondary mineral macronutrients (2), and Pb (3). The first and second groups are mainly conditioned by PC1, whereas the third group (Pb) is mainly conditioned by PC2. However, Mg and Cd are conditioned by PC1, demonstrating that those parameters have also a high affinity to PC2. Due to distribution of variables, it was found that the increase of Pb is the main conditioning factor of the second growing season with a simultaneously reduced amount of other elements in the aboveground plant organs, which corresponds to the results visible on the spider charts.

Among *M.* x *giganteus* samples supplemented by fertilizers in the growing season 2014, the highest HMs accumulation was observed for plants treated with NPK while the lowest accumulation ability was in *M.* x *giganteus* untreated and treated with an inoculum. For *S. pectinata* in the growing season 2014, the highest heavy metals (mostly Zn) accumulation was observed for plants treated with the inoculum; however, in the case of NPK treated and control plants, the same or lower values were observed compared to the inoculated plants. In *M.* x *giganteus* samples collected at the end of the first growing season, the highest primary mineral macronutrients content was observed for plants treated with the inoculum, whereas significantly lower values (except N in control plants, which was statistically the same) were observed for untreated and NPK-treated plants. The lowest results for N and K were found in plants treated with NPK fertilizer. Among *S. pectinata* plants in the growing season 2014, the highest accumulation of primary macronutrients was observed for plants treated with NPK fertilizer. Moreover, equally lower values of these elements were found for *S. pectinata* control plants and those treated with microbial inoculum in comparison with an NPK treatment.

For *M.* x *giganteus* plants in the growing season 2014, Mg accumulation was the highest for all plants treated with NPK and microbial inoculum, with statistical significant difference to the control. Accumulation of Ca in the growing season 2014 was the same for *M.* x *giganteus* treated with an inoculum and the control. Moreover, NPK treatment caused a lower Ca accumulation when compared to control. Accumulation of Mg among *S. pectinata* plants was the same for each experimental option. Different observations were found for Ca, where *S. pectinata* treated with NPK and with microbial inoculum had slightly lower Ca plant concentration in comparison to the control. Water content after the first growing season was higher for *M.* x *giganteus* when compared to *S. pectinata*; however, in both cases, treatments had no significant influence on this parameter.

After the second growing season, trends for HMs and primary mineral macronutrients were similar to those observed in the first one. Among secondary macronutrients accumulation, Mg and Ca showed different trends in the growing season of 2015 when compared to the growing season of 2014. *M.* x *giganteus* plants cultivated with NPK and inoculum fertilizers showed higher Mg concentration in the aboveground organs when compared to the control. Among the treatments, the highest Mg concentrations were observed for inoculated *M.* x *giganteus* plants. In the growing season 2015, the highest Ca concentration was observed for NPK-treated *M.* x *giganteus*; however, lower and statistically equal values were reported for plants treated with the inoculum and the control plants. Among *S. pectinata* plants, tendencies in accumulation of HMs and primary macronutrients during the growing season of 2015 were the same when compared to the growing season of 2014. Element contents were the same independent of the treatment, with the only exception of Pb, which was significantly lower in comparison to the control. Concerning the secondary mineral macronutrients, accumulation in the second growing season showed that treatments did not affect Ca and Mg accumulation. After the growing season 2015, water content showed the same trends as in the growing season 2014.

This section corresponds to an overall pattern trend of the three groups: HMs, primary macronutrients, secondary macronutrients and water content. Statistical analysis of values used for spider chart construction, specific for the described parameters is presented in Table 2. In addition to the presented analysis, three-way ANOVA was performed to detect which factors, i.e., plant, year, fertilization, drove changes in the analyzed parameters (Table 3). Undoubtedly, factors corresponding to the plant species and the year of cultivation caused meaningful differences among all the investigated parameters. Fertilization affected significantly only Mg and Zn concentration in the investigated plant biomass. While considering a combined effect of the factors, i.e., plant × year × fertilization, the significant changes appeared among N, Pb, and Zn concentrations in the plant biomass.

**Table 2:** Matrix shows statistical significant differences among analyzed parameters presented on spider charts (Fig. A.3, B.2)

	Parameters								
	WC	Mg	Ca	P	K	N	Pb	Cd	Zn
Variants									
M-C/14	a	cd	a	bc	b	a	a	ab	b
M-NPK/14	a	a	b	ab	b	b	c	a	a
M-INC/14	a	ab	a	a	a	a	a	ab	b
M-C/15	c	f	bcd	d	def	d	b	d	cde
M-NPK/15	c	e	bc	e	ef	d	a	bc	c
M-INC/15	c	d	bc	de	ce	d	ab	c	cd
S-C/14	b	ef	a	c	cd	c	c	e	cd
S-NPK/14	b	ef	bc	c	bc	c	c	e	d
S-INC/14	b	f	c	c	cd	c	c	e	c
S-C/15	d	ef	d	de	f	d	a	e	e
S-NPK/15	d	ef	cd	e	f	d	b	de	e
S-INC/15	d	ef	cd	de	f	d	ab	de	d

A lower case letter (a, b, c, d, e, f—where “a” corresponds to the highest value and “f” to the lowest) denotes significant differences among plants elements and water content taken from different plots at *P* ≤ 0.05 according to Fisher LSD test. Every measurement was performed in five replicates (*n* = 5)

*M Miscanthus* x *giganteus*, *S Spartina pectinata*, *C* control, *NPK* NPK fertilized plants, *INC* microbial inoculated plants

**Table 3:** Results of three-way ANOVA testing the effects of plant (P), year (Y), and fertilization (F) and combine effects of those factors on different biomass composition parameters

Factor	*F* value								
	Water content	Mg	Ca	N	P	K	Pb	Cd	Zn
Plant	145.27***	195.43***	25.02***	53.32***	18.21***	34.95***	11.56**	183.54***	139.41***
Year	554.99***	12.08**	29.60***	376.22***	241.09***	102.95***	55.57**	12.64***	114.22***
Fertilization	0.19	8.05***	0.45	0.75	0.88	2.28	3.09	2.51	3.52*
P x Y	1.01	31.04***	2.18	34.70***	5.47*	5.54*	15.56**	18.52***	35.91***
P x F	0.52	6.75**	0.17	2.37	0.42	6.70**	1.03	1.07	10.51***
Y x F	0.56	2.355	4.34*	2.04	5.36**	0.16	1.30	1.27	0.60
P x Y x F	0.64	0.566	1.32	4.82*	2.39	1.51	8.30***	0.99	4.51*

*P < 0.05, **P < 0.01, ***P < 0.001; significant values (*P* < 0.05)

### Plant biomass production

**Fig. 5:**
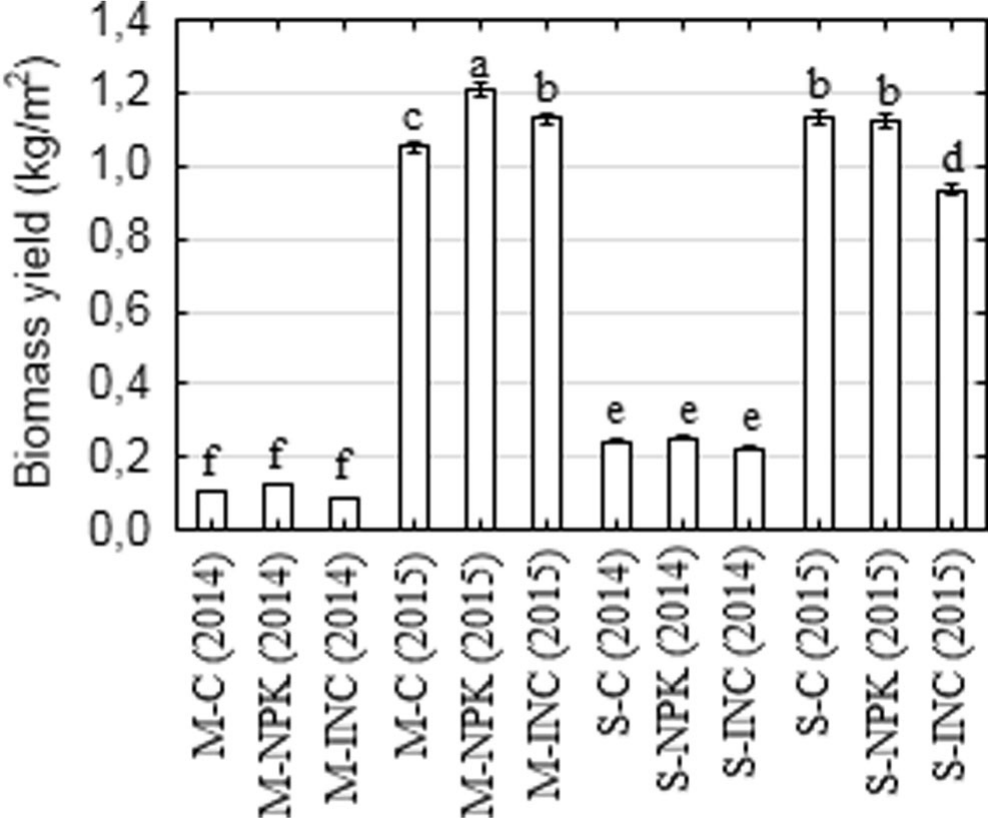
Biomass yield of *Miscanthus* x *giganteus* and *Spartina pectinata* after 2014 and 2015 growing seasons Data are means ± SE (*n* = 5). A lower case letter (a, b, c, d, e, f) denotes significant differences among biomass yield obtained from different plots at *P* ≤ 0.05 according to the Fisher LSD test. *M Miscanthus* x *giganteus*, *S Spartina pectinata*, *C* control, *NPK* NPK fertilizer, *INC* microbial inoculum

After the growing season 2014, higher biomass yield was found for *S. pectinata* in comparison to *M.* x *giganteus* (by about 126%). Moreover, the treatments did not influence the yield. A visible effect was observed for treatments in *M.* x *giganteus* as well as in *S. pectinata* plants after the growing season 2015. It inferred that the inoculum had a different impact on both species. *M.* x *giganteus* biomass yield was increased by 14%, while *S. pectinata* biomass decreased by 26% when compared to the control. NPK treatment did not affect *S. pectinata* biomass production; however, it increased biomass yield by 18% in NPK-treated *M.* x *giganteus* (Fig. 5).

### Plants visual observation

Plant photography is presented in Fig. S1. Beside visual differences in the height and stem density of individual plants for both growing seasons, there is a visible difference in the surface of the withered zones observed for *M.* x *giganteus* plants as well as for *S. pectinata*, which is larger at the end of the second growing season (beginning in September). Moreover, the withering effect of both species was not determined by the application of fertilizers. The results presented in the “Heavy metals and mineral macronutrients plant concentrations (spider charts and PCA)” section together with the photographic documentation corresponding to this section indicate hasted senescence in the 2015 growing season, when compared to 2014. Plant senescence occurred earlier due to drought conditions during the 2015 growing season.

## Discussion

It was found that *M.* x *giganteus* biomass production seems to be more efficient (0.5–4.4 kg m^−2^ d.w.) when compared with *S. pectinata* (0.4–1.8 kg m^−2^ d.w.) (Lewandowski et al. 2003). In the current study, after the second growing season, the obtained biomass yield was in the range of the abovementioned values for both species. However, the yield of the first year biomass was lower due to plant acclimatization (Borkowska and Molas 2013). A higher biomass yield for *S. pectinata* in the first growing season and similar for both species during the second could be attributed to higher concentrations of HMs on plots where *M.* x *giganteus* was cultivated. Those findings could be additionally supported by the fact that younger plants grown on soil contaminated with HMs are more sensitive to stress associated with an excess level of these metals than older plants, which could resulted in a lower *M.* x *giganteus* yield when compared to *S. pectinata* (Kocoń and Jurga 2017).

Interestingly, it was found that only *M.* x *giganteus* showed a positive response in biomass yield to the applied fertilizers. Contrary results were obtained for *S. pectinata*, confirmed by a similar yield obtained after NPK chemical fertilization and lower yield after microbial inoculation when compared to control. Monti and Zegada-Lizarazu (2016) reported that during 16 years of *Arundo donax L.* cultivation supplemented with a moderate dose of N fertilizer, no significant effect on the obtained biomass yield was observed. Application of a higher dose of N fertilizer resulted in a significantly higher biomass yield only in a few growing seasons. This phenomenon reported by Monti and Zegada-Lizarazu could indicate that the response of grasses to fertilization depended not only on the dose of the fertilizer but the growing season as well. A lower biomass yield obtained for inoculum-treated *S. pectinata* plant may imply competition between indigenous microorganisms and those present in inoculant were taken into consideration. Vázquez et al. (2000) reported that after inoculation, modifications of the microbial community structure and ecology were found; however, the quality and quantity of these modifications could be unexpected especially for plant growth and development.

Despite the fact that the described experiment was not carried out additionally on an uncontaminated soil, the application of fertilizers resulted in a changed pattern of the spider charts. Interestingly, while a spider chart pattern covered a larger area conditioned by primary macronutrients simultaneously, the smaller area conditioned by HMs was covered. The results seem to confirm our hypothesis, that HMs can decrease primary mineral macronutrients in plants. Patterns of reducing primary macronutrients due to the increase of HMs are visible for *M*. x *giganteus* as well as for *S. pectinata* in both analyzed growing seasons. In this case for both plants species in the HMs spider charts area, Zn seemed to be the dominating factor in conditioning the area size within the first growing season, which was also revealed by three-way ANOVA, while Pb seemed to condition the second season. There are reports which indicate HMs influence on mineral macronutrients accumulation in different plant species (Goncalves et al. 2009; Naz et al. 2013); however, there are also reports which show no effect of HMs on plant mineral macronutrients composition (Zhang et al. 2002; Garedea-Torresdey et al. 2004). This influence is dependent on the plant species, cultivar, concentration of HMs in soil (medium), time of exposure, and the environment or medium in which plants were cultivated (Zhang et al. 2002; Goncalves et al. 2009). Additionally, there is shortage of papers showing the influence of HMs on primary mineral macronutrients in *S. pectinata* (Helios et al. 2014) and *M.* x *giganteus* (Nsanganwimana et al. 2016).

The general spider chart pattern overview indicated that *M.* x *giganteus* demonstrated a better ability to accumulate heavy metals, as well as mineral macronutrients. In fact, in the initial soil, higher values for those elements on *M.* x *giganteus* plots were observed, which undeniably influenced their accumulation in plants. After the second growing season, the differences in the overall accumulation between species were not as high as in the first one. Korzeniowska and Stanislawska-Glubiak (2015) reported that in the microplot experiments with artificially contaminated soil up to the depth of 0–30 cm, *S. pectinata* had a better ability to accumulate Zn in aboveground organs in the second year after the experiment establishment than *M.* x *giganteus*. Although an opposite phenomenon was observed in our research, differences could be explained by the lack of soil homogeneity when considering differences between plant species.

Lead concentration presented on the spider charts and the PCA analysis, after the second growing season, showed inconsistent patterns throughout the year. An increased Pb concentration was observed for *S. pectinata* while for *M.* x *giganteus* it remained unchanged, with the exception of NPK-treated plants, where it was increased. Lead accumulation in aerial parts depended mainly on the age of the leaves. It has been found that the highest concentration of Pb occurred in the senescing leaves while the lowest was observed in the young ones (Islam et al. 2008). This can be associated with Pb’s ability to form complexes with anionic sites associated with pectic substances within the cell wall (Donnan-free space) (Singh et al. 1997). Such a phenomenon corresponds with the obtained results, where the relocation of Pb was limited and stayed on the same level, regardless to the progression of senescence each year.

*M.* x *giganteus* and *S. pectinata* biomass can be processed in many ways depending on the stage of the plants growth and the availability of the processing technologies. There are many reports which describe different approaches to energy production from biomass produced from these plants. Most of the technologies are connected with biomass thermo-chemical conversion (McKendry et al. 2002a) and typically require dry biomass (low moisture content). For that purpose, plants are usually harvested at late winter or early spring (McKendry et al. 2002b; Robson et al. 2012). However, there are also processes which prefer green (high moisture content) biomass (McKendry et al. 2002a).

In case of perennial grasses which acquire mineral nutrients to the rhizomes, plant senescence is the most important factor that determines the time of biomass harvesting. It was reported that in plants harvested at the beginning of autumn, the allocation process of elements is weaker in the following year due to interference (Kiesel and Lewandowski 2017). The senescence process is not constant for plants every year; it can be hasted or delayed by many factors including environmental (Thomas 2013; Lim et al. 2007) and plantation age in the field condition (M. Pogrzeba, personal communication). In the presented study, time varying senescence is showed on the spider charts and the PCA analysis. After the second growing season, a considerably lower concentration of the mineral macronutrients and water content was observed when compared to the first one, which could correspond to the hasted allocation of nutrients. To coop with different senescence time, which could depend on the weather conditions, there is a need of a method that would be suitable to determine the development stage of a plant. For that purpose, portable chlorophyll content meters could be used as a senescence indicator, as previously reported (Mos et al. 2013).

## Conclusion

Fertilizers application had a positive effect on *M.* x *giganteus* biomass yield, while NPK chemical fertilizer and microbial inoculum had no effect and caused lower yield of *S. pectinata*. Macronutrients concentration (particularly N and K) were affected by higher heavy metals concentration in shoots resulted from different fertilization regimes. Relocation of Pb was limited and stayed on the same level, regardless to the progression of senescence each year. Drought observed during the 2015 growing season caused hasted senescence when compared to the 2014 growing season.

## Electronic supplementary material

Fig. S1 (DOCX 3907 kb)
