# Supplementary material for: Macroelements and heavy metals content in energy crops cultivated on contaminated soil under different fertilization—case studies on autumn harvest
Source: Environ Sci Pollut Res Int. 2018 Feb 16;25(12):12096–106. doi: 10.1007/s11356-018-1490-8 (PMC5940723; doi:10.1007/s11356-018-1490-8)

**Supplementary Figure 1.** Photography documentation of *Miscanthus* x *giganteus* (A-F) and *Spartina pectinata* (G-L) at the begining of September 2014 (A-C and G-I) and 2015 (D-F and J-L)


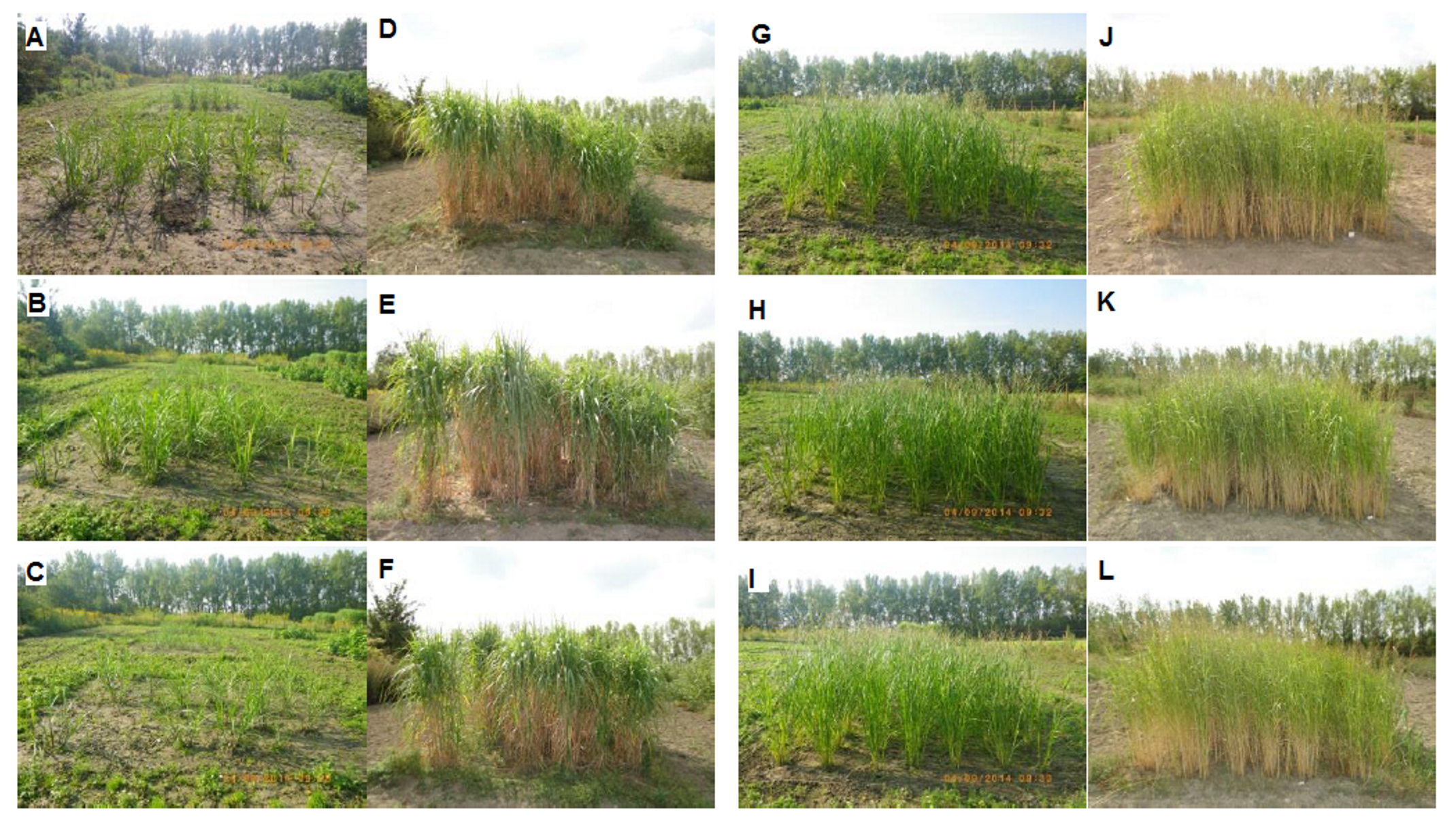

Supplement: Supplementary file 1 — (DOCX 3907 kb) [file 11356_2018_1490_MOESM1_ESM.docx]
